# An Innovative Communication Approach to Mitigate Suffering Among COVID-19 Patients and Their Families: An Experience From Oman

**DOI:** 10.3389/fpubh.2021.664007

**Published:** 2021-07-19

**Authors:** Harith Al Harthy, Jehan Al Fannah, Hasina Al Harithi, Sitwat Usman Langrial, Salah T. Al Awaidy

**Affiliations:** ^1^Royal Hospital, Ministry of Health, Muscat, Oman; ^2^Department of Information Systems and Technology, Sur University College, Sur, Oman; ^3^Office of Health Affairs, Ministry of Health, Muscat, Oman

**Keywords:** Coronophobia, Lasswell's communication model, innovative communication, design principles for communication, Oman

## Introduction

Healthcare systems across the globe continue to counter the COVID-19 pandemic with the need for coherent and effective treatment posing a significant challenge. “Coronophobia” is a new term that was recently published describes various negative psychological signs that are assumed to be an outcome of the fear of COVID-19 infection (1). The prevalence of Coronophobia is being associated with high infectivity and mortality rates (1). People's concerns about quarantine, internment, and social isolation have further aggravated the situation. Uncertain and distorted information in the media has made the situation worse (1).

COVID-19 has developed into a unique pandemic that is accompanied by complex communication challenges. The details of COVID-19 pathophysiology and standards treatment plans are not yet clear hence making it even more important to have functional and continuous communication with patients and their families at regular intervals for information sharing and better decision making. However, the restriction of face-to-face communication as a precaution to prevent further spread of the infection has made effective communication even more difficult.

Solitude and controlled visits to COVID-19 patients to minimize the risk of community transmission have obstructed communication among healthcare professionals, with the patients and their families. The psychological effect of social isolation and quarantine on patients has negatively predisposed their prognosis and psychotherapy (2, 3), while their families continue to experience anxiety. Lack of communication has left healthcare workers emotionally distressed, as emotional support for families by healthcare workers leading to poor decision-making that is integral to professional medical care and should not be affected (2).

While we struggle to establish natural and real time communication between patients and their families, one way forward is to utilize telecommunication instruments to minimize negative impact of disrupted communication channels (3, 4). Oman is facing similar challenges as the rest of the world when it comes to lack of communication during isolation and quarantine. Oman provides healthcare to its citizens and residents with COVID-19 at no charge, however language barriers and poor communication have increased and led to challenges for effective communication.

The Royal Hospital is a public tertiary hospital in Oman with 1,056 beds. During the COVID-19 pandemic, its Intensive Care Unit's (ICU) capacity was forced to increase from 16 adult beds to more than 50 beds. The ICU's bed occupancy rate was over 100%, with several multiple COVID-19 ambulatory care wards expansions to manage an exponentially growing COVID-19 cases. Patients with moderate symptoms were admitted to ambulatory institutions while those with serious symptoms were admitted to the ICUs.

## Barriers to Access Care

The Omani citizens represents 56% of the total population (~4.6 million) and the non-citizen resident population represents the remaining 44% (~2 million) (5). Most non-citizen (5) are males from India, Bangladesh, or Pakistan who were served mostly by private healthcare institutions. During the early phase of the pandemic, most of COVID-19 critical patients regardless of their nationality were diverted to the public sector.

Access criteria were critical during the early stage of the COVID-19 pandemic when the daily occurrence of infection among the non-citizen residents was much higher than among the Omani citizens (6). For many of the non-citizen population, access, and affordability of the intensive care high costs was not clear and might have prevented some of them from seeking help in a timely manner. In a short time span and in very unconventional situation, with the complex demographics and regulatory framework, designing a healthcare system with rightful services access that can meet the diverse nature of the population might be yet another challenge.

## Barriers to Communication

From a patient's viewpoint, a hospital admission can at times be overwhelming especially for those who are alone without family, visitors, or interactions with caregivers following the isolation protocols.

Improving communication plans between healthcare workers, patients, and their families is of utmost significance with the communication needs of all stakeholders meriting top priority and attention. The stakeholders' triad results in multiple areas of conceivable communication gaps that require emphasis when designing a communication plan for COVID-19 patient care. Apart from the patients, the other two stakeholders (healthcare professionals and families) have their own internal communication gaps that need to be focused. The significance of overcoming these potential communication gaps are available in literature, clinician-patient-family communication pathways triangle (7).

When the first wave of COVID-19 hit Oman, numerous complaints were received and recorded by the Royal hospital from patients, their families and even healthcare professionals about an almost complete lack of communication between patients and their families as the guidelines from the Ministry of Health relating to isolation and quarantine were being strictly followed. Strict quarantine measures helped us identify instances where negative psychological behaviors and attitudes were evident among the patients, their families and even health care professionals.

## General Design Principles for Developing Communication Solutions

**Figure 1:**
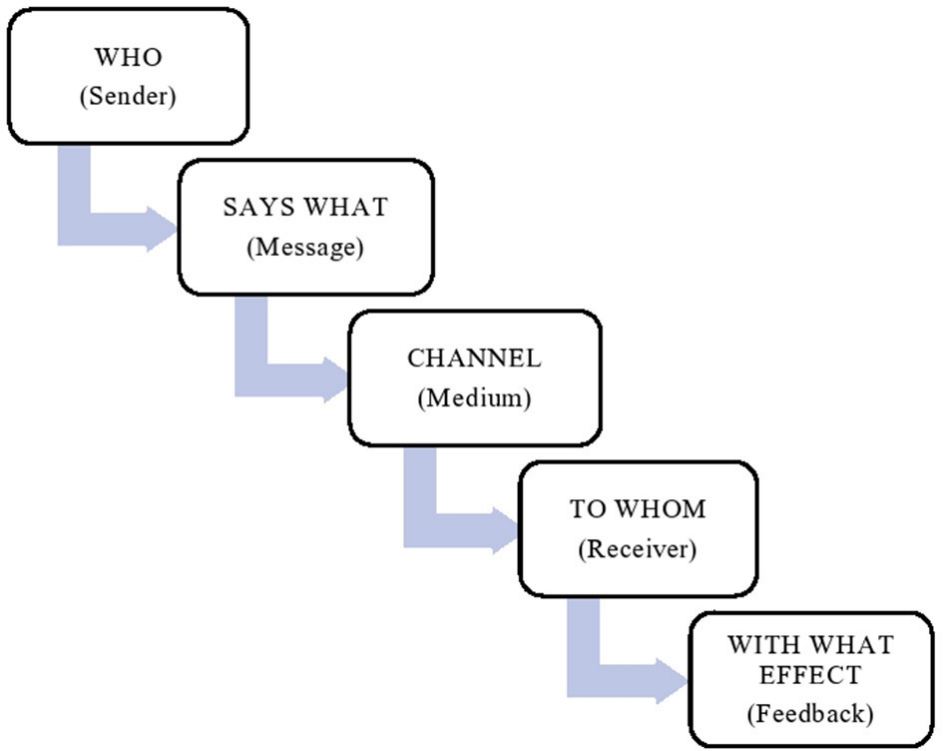
Components of Lasswell's communication model (8, 9).

We used Lasswell's Model (8, 9) (Figure 1) to assist in information gathering during multi-stakeholder meetings. The Model describes five important communication elements, who, says what, in which channel, to whom and with what effect. This model is also called a “linear model of communication,” “unidirectional” processes or “action model” because it describes a one-way process within a communication. The model can be used as an analysis tool for evaluating the communication process to decide areas needing improvement.

The above tool from Lasswell's model helped us in analyzing the existing communication gap and enhanced the communication plan between different stakeholders.

### Solution for Communication Gap 1: Healthcare Workers to Families

A unique situation for COVID-19 patients was that upon their admission either directly from the Emergency Department (ED) or as referred from another health institution to the hospital, they underwent quarantine directly with other family members of the patients being placed under home quarantine too. This resulted in absent or very limited access information for patient's families to communicate.

During the first step of the admission process, it was observed that contact details for the patients' families were, in many instances, either not recorded, or not updated in the health records of the patient complicating the clinical teams' ability to contact the family.

To solve missing contact details, a form was devised and integrated with the electronic medical records of the patients. It was designed to be filled out as the first step of the admission process upon accepting a referral or admitting a patient from the ED. The form captured the details of 3 principal family contacts together with their mobile phone numbers that has WhatsApp® application installed and enabled.

A staff of the patient's registration team in the hospital was responsible to create a dedicated WhatsApp® group assigned for every COVID-19 patient. The WhatsApp mobile number accounts of key staff who were responsible to receive, send, manage, and monitor the communication plan (non-clinical) between the family and healthcare professionals were added to the group.

### Communication Gap 2: Patient to Family

Patients who were admitted to non-ICU wards, or were not on ventilators, could communicate with their families using their own mobile phones. The isolation areas of the hospital had strong network coverage. For patients who did not have mobile phones or were unable to use them, the hospital provided them with network-enabled i-Pads® to facilitate communication.

### Solution for Communication Gap 3: Healthcare Worker and Patient

The patient experience team focused on enhancing communication between patients and their healthcare professionals. Several training sessions for all the staff caring for COVID-19 patients on communication skills were conducted, including a thorough explanation of the 8 principles of person-centered care outlined in “Picker's eight principles” (10).

Picker's eight principles focuses on prompt and timely access to health care, smooth transitions in a continual care plan, access to information and support needed for self-care, involvement of the patients in decision making, involvement of the family with the care providers, attention to the physical and environmental needs, support, and treatments services that are effective and built on mutual trust and respect. Furthermore, a checklist was developed to ensure each and every staff adhere to the communication plan.

### Implications and Recommendations Based on Our Care Experience Efforts

The Lasswell's Communication model allowed us to identify and analyze communication gaps in the ongoing pandemic. With this approach, we were able to rapidly test and explore many patients suffering from communication gaps and employee-specific processes, bringing teams together, using technologies and means in new ways and identify simple solutions to address complex issues.

## Conclusion and Future Research

This short communication highlights how Coronophobia is contributing to high infectivity and mortality rates. It also sheds light on the negative effects of social isolation and abundantly available misleading information. Having identified barriers to access and communication and applying the preliminary design principles, we observed a significant number of reductions in complaints received at the Royal Hospital and hence we are confidently proposing preliminary design principles for improved and effective communication solutions. We strongly believe that our proposed solutions will enhance communication effectiveness, teamwork and better decision-making when dealing with patients suffering from COVID-19. Future research is indispensable to enhance communication and research is called for to address on reducing communication gap between patients, families, and healthcare professionals.

## Author Contributions

All authors listed have made a substantial, direct and intellectual contribution to the work, and approved it for publication.

## Conflict of Interest

The authors declare that the research was conducted in the absence of any commercial or financial relationships that could be construed as a potential conflict of interest.

## References

[1] Dubey S Biswas P Ghosh R Chatterjee S Dubey MJ Chatterjee S. Psychosocial impact of COVID-19. Diabetes Metab Syndrome. (2020) 14:779–88. 10.1016/j.dsx.2020.05.035 PMC7255207 32526627

[2] Rose L Cook A Casey J Meyer J. Restricted family visiting in intensive care during COVID19. Intensive Crit Care Nurs. (2020) 60:102896. 10.1016/j.iccn.2020.102896 32601012 PMC7261454

[3] Akgün KM Shamas TL Feder SL Schulman-Green D. Communication strategies to mitigate fear and suffering among COVID-19 patients isolated in the ICU and their families. Heart Lung. (2020) 49:344–5. 10.1016/j.hrtlng.2020.04.016 32451114 PMC7196381

[4] Negro A Mucci M Beccaria P Borghi G Capocasa T Cardinali M. Introducing the video call to facilitate the communication between health care providers and families of patients in the intensive care unit during COVID-19 pandemia. Intensive Crit Care Nurs. (2020) 60:102893. 10.1016/j.iccn.2020.102893 32576488 PMC7247985

[5] De Bel-Air F. Demography, Migration, and the Labour Market in Oman. (2018). Available online at: https://gulfmigration.org/media/pubs/exno/GLMM_EN_2018_07.pdf

[6] Khamis F Al-Zakwani I Al Naamani H Al Lawati S Pandak N Ba Omar N. Clinical characteristics and outcomes of the first 63 adult patients hospitalized with COVID-19: an experience from Oman. J Infect Public Health. (2020) 13:906–13. 10.1016/j.jiph.2020.06.002 32546437 PMC7832725

[7] Shapiro J. Using triangulation concepts to understand the doctor-patient-family relationship. Families Syst Health. (2001) 19:203. 10.1037/h0089544

[8] Wenxiu P. Analysis of new media communication based on Lasswell's “5W” model. J Educ Soc Res. (2015) 5:245–50. 10.5901/jesr.2015.v5n3p245

[9] Sapienza ZS Iyer N Veenstra AS. Reading Lasswell's model of communication backward: three scholarly misconceptions. Mass Commun Soc. (2015) 18:599–622. 10.1080/15205436.2015.1063666

[10] Principles of Person Centred Care: Picker. Available online at: https://www.picker.org/about-us/picker-principles-of-person-centred-care/ (accessed December 12, 2020).

